# Clinical Characteristics and Surgical Procedures for Children with Congenital Membranous Cataract

**DOI:** 10.1155/2017/2370969

**Published:** 2017-11-23

**Authors:** Jingji Long, Daoman Xiang, Zheng Guo, Lihong Chen, Feng Chen, Jianxun Wang, Wanhua Xie, Shiping He

**Affiliations:** ^1^Department of Pediatric Ophthalmology, Guangzhou Children's Hospital and Guangzhou Women and Children's Medical Center, Guangzhou Medical University, Guangzhou, Guangdong 510230, China; ^2^Department of Pediatric Ophthalmology, Wuhan Children's Hospital, Wuhan 430015, China; ^3^Department of Ophthalmology, Shenzhen Longhua New District Central Hospital, Shenzhen, Guangdong 518110, China

## Abstract

**Objective:**

In a group case series, the clinical characteristics of congenital membranous cataract in children were studied to establish a system of classification and determine the surgical method suited for each type.

**Methods:**

Children (18 eyes) with congenital membranous cataract were examined by slit lamp, ultrasound biomicroscopy, and operating microscopy to classify cataracts. The clinical characteristics of congenital membranous cataract and its feature related to the surgical method were analyzed.

**Results:**

Five major types of congenital membranous cataracts were classified. All of the surgeries were successful. Anterior and posterior capsulorhexis was performed using Klöti RF capsulotomy tips. The capsular flap was removed, and anterior vitrectomy was performed using a vitrectomy cutter. Postoperative complications included posterior capsule opacification in 16.7% of the patients.

**Conclusion:**

Ultrasound biomicroscopy was used successfully to classify congenital membranous cataracts prior to surgery. Anterior and posterior capsulorhexis was performed using Klöti RF capsulotomy tips, and capsulectomy was performed using a vitrectomy cutter. These were effective techniques and should be considered for congenital membranous cataract removal surgery. This trial is registered with registration number chiCTR-OOC-17010913.

## 1. Introduction

Membranous cataract was first reported in 1833 [1, 2] as a congenital disorder in which the lens is flattened with little or no fiber cells. The condition is usually diagnosed during childhood. Patients usually do not have other ocular diseases such as glaucoma, uveal inflammation, microphthalmos, ocular tumor, ocular trauma, or systemic disease. The etiology of congenital membranous cataract is unclear, but *Rubella virus* infection during the early part of the pregnancy could be a contributing factor [3]. In most studies, the subjects have been adults [4–8], but a few have involved children [8, 9].

Slit lamp examination is the traditional and common method for observing most cataracts. However, because of capsular opacity, slit lamp examination may not be used to diagnosis most congenital membranous cataracts. Ultrasound biomicroscopy (UBM) was first used to diagnose the membranous cataract of a 3-month-old baby [9]. UBM could provide a precise image of the ciliary body and helped to predict possible complications during surgery [9]. UBM has been used subsequently to observe a congenital membranous cataract [10], but a systematic classification of congenital membranous cataract has not been established.

Several surgical procedures for congenital membranous cataract have been described (Table 1). Continuous curvilinear capsulorhexis is difficult for membranous cataracts, and the membrane must be cut using scissors. A cystitome was used to make a radial nick extending beyond the central fibrous plaque and then to raise the capsule flap and complete the capsulorhexis. The fibrotic plaque adherent to the anterior capsule was removed at the same time. Because membranous cataractous lenses are thin, fibrous, and sticky, they are difficult to remove using only aspiration, and so routine pars plana lensectomy was tried [9]. Pars plana lensectomy is not necessarily the safest surgical procedure, but it was found that a vitreous cutter was able to cut and aspirate the lens material without any complications [9]. More recently, a capsulotomy and excision of the membranous capsule with vitreoretinal scissors were successful [11]. Ehrlich [3] reported a bilateral case and described how, on needling the remaining membrane, no cortical matter was found and a clear pupil was achieved.

This study reviewed the clinical characteristics of congenital membranous cataract in children to classify the different types of the disorder and the surgical method best suited for each type. The effectiveness of capsulorhexis using a Klöti RF capsulotomy tip and capsulectomy using a vitrectomy cutter is highlighted.

## 2. Materials and Methods

Between January 2010 and May 2015, we enrolled 12 patients (18 eyes) who had received a diagnosis of congenital membranous cataracts in the Department of Ophthalmology of Guangzhou Women and Children's Medical Center (Table 2). The patients did not have any other ocular diseases, including glaucoma, uveal inflammation, microphthalmos, ocular tumor, ocular trauma, or systemic disease. The Institutional Review Board/Ethics Committee of Guangzhou Children's Hospital and Guangzhou Women and Children's Medical Center, Guangzhou Medical University, approved this study. The study was performed in accordance with the ethical principles described in the Declaration of Helsinki. All patients enrolled in the study agreed to participate, met the inclusion criteria, and signed an informed consent agreement before any procedures were performed. The consent procedure was approved by the Committee of Guangzhou Children's Hospital and Guangzhou Women and Children's Medical Center, Guangzhou Medical University.

### 2.1. Examinations

Patients were informed in detail about the natural course of the disease, the different treatment options, and the possible complications. Prior to cataract surgery, patients were given a comprehensive physical examination, including a medical examination, blood test, chest X-ray, and an electrocardiogram test, and were examined for associated risk factors such as diabetes, hypertension, cardiovascular disease, and bleeding disorders. All the patients underwent clinical examination by a slit lamp and operating microscope. Six patients had a UBM examination both before and after surgery. One patient had a UBM examination after surgery only.

#### 2.1.1. Slit Lamp

Patients took chloral hydrate prior to the examination by a portable slit lamp (SL-15, Kowa, Japan), and slit lamp (SL-D7, TOPCON, Japan) observations were made with the patients' pupils dilated.

#### 2.1.2. UBM

UBM (Quantel Medical, MT, USA, 50 MHz probe) examination was performed by an experienced technician. The patients took chloral hydrate prior to the examination, while supine. A nurse helped the technician to steady the patient's head by hand.

#### 2.1.3. Observation by Operating Microscope

The surgeon characterized the opacity of the lens prior to cataract surgery, with the patient under general anesthesia and the patient's pupils dilated.

### 2.2. Surgery

All surgeries were performed by the same surgeon (author D.X.) with the patients under general anesthesia. The initial approach was a 3.2 mm scleral tunnel, 2 mm posterior to the limbus. Sodium hyaluronate was injected into the anterior chamber, and capsulorhexis was performed with a Klöti RF capsulotomy tip and a cataRhex, Swisstech phacoemulsification device (Oertli, Berneck Switzerland). Capsulectomy and anterior vitrectomy were performed using a vitrectomy cutter (cataRhex, Swisstech phacoemulsification device, Oertli, Berneck Switzerland). Anterior capsulorhexis was performed with a Klöti RF capsulotomy tip. The lens material was removed with irrigation, aspiration, or both (I/A) (cataRhex, Swisstech phacoemulsification device, Oertli, Berneck Switzerland). Posterior capsulectomy was performed using a Klöti RF capsulotomy tip. The scleral incision was closed with 10.0 nylon sutures. All patients received 2 mg subconjunctival tobramycin and dexamethasone at the end of surgery. Four patients needed intraocular lens (IOL) implantation. All patients were observed from 2 to 48 months (median 25 months) after surgery.

## 3. Results

The 12 patients (18 eyes) with congenital membranous cataracts were referred to our hospital with a white pupil. Their parents reported a white pupil since birth. Family history was unremarkable. Slit lamp examination revealed a clear cornea and anterior chamber in both eyes. All cataract removal surgeries were performed successfully. Posterior capsule opacification (PCO) was a postsurgical complication that occurred in 16.7% of the patients. PCO was treated as a planned secondary operation.

Congenital membranous cataracts were confirmed by UBM in 6 patients (9 eyes) prior to surgery. Another 6 patients (9 eyes) were not examined by UBM and could not be classified until the capsule was cut during the surgery.

**Figure 1:**
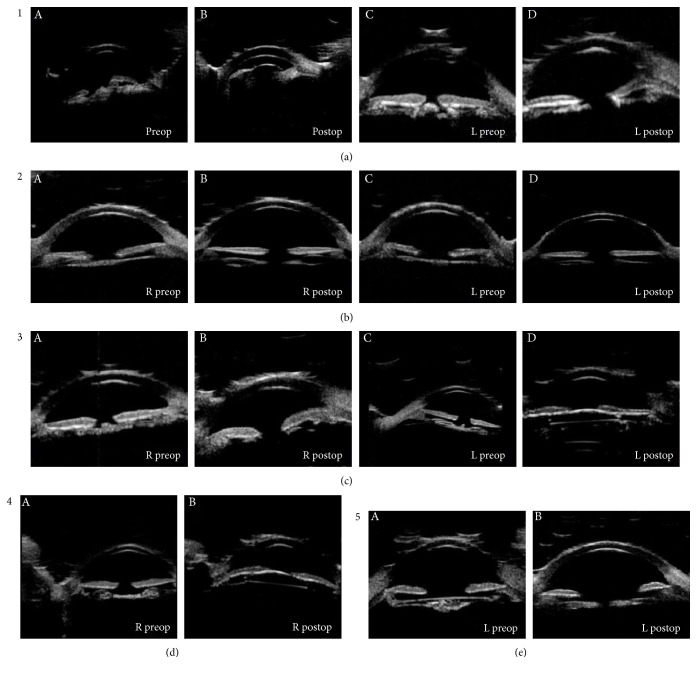
Ultrasound biomicroscopy images for different classifications of cataract. UBM type 1 (a): (A, C) the anterior segment appeared as a thick high echoic area (like pizza) in the lens area (arrow). (B, D) An anechoic area in the central axis is visible after cataract surgery. A curved high echo in the shallow anterior chamber is visible (arrow: inflammatory membrane).UBM type 2 (b): (A, C) a linear high echo area in the lens of the anterior segment was seen (arrow). (B, D) An anechoic area in the central axis in the anterior segment was visible. UBM type 3 (c): (A, C) a high echoic area like a flying saucer was observed (arrow). (B, D) An anechoic area in the central axis was observed after the cataract surgery. UBM type 4 (d): (A) a high echoic area like a dumbbell was visible in the right eye. (B) A curved high echo in central axis after IOL implantation was visible. UBM type 5 (e): (A) A fusiform high echoic in the lens area was observed (arrow). (B) An anechoic area was observed along the central axis after cataract removal surgery.

UBM imaging was used to classify five types of UBM congenital membrane cataracts as follows (Figure 1): type 1—the lenses appear thickened and pizza-shaped. Type 2—the lenses appear linear. Type 3—the lenses appear as flying saucer-shaped. Type 4—the lenses appear as dumbbell-like. UBM type 5—the lenses appear fusiform.

**Figure 2:**
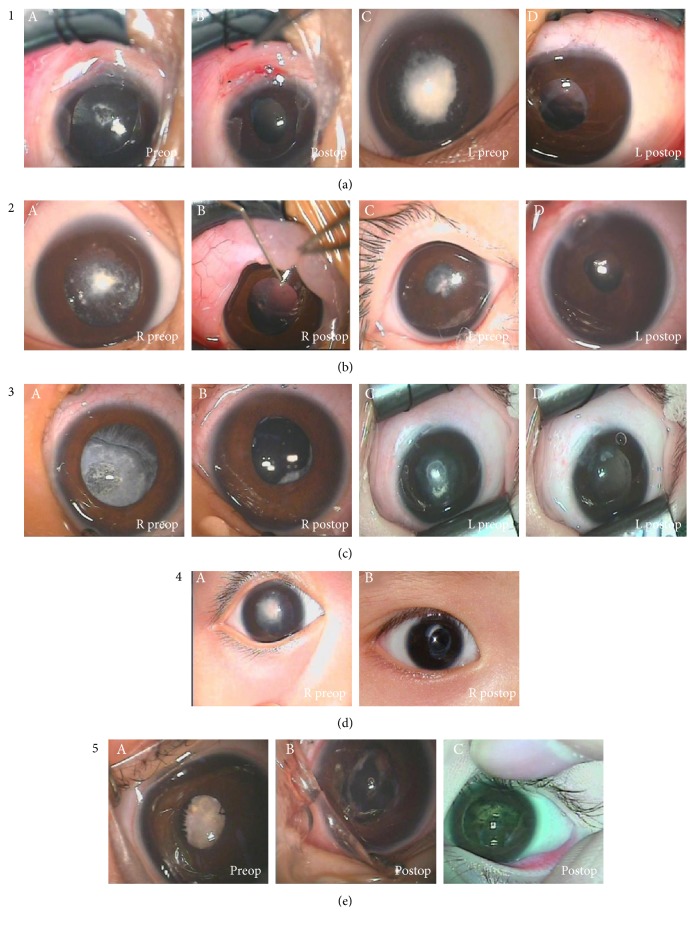
Operating microscope photographic images for different classifications of cataract. OM-type 1 (a): the cortex was partially absorbed. (A, C) The pupil was covered by a daisy-like white opacity in the central region with a semiclear area in the peripheral region before cataract surgery. (B, D) A clear visual axis appeared in the central pupil after cataract surgery. OM*-*type 2 (b): the cortex was mostly absorbed (missing). (A, C) The pupil was covered by a white central opacity before cataract surgery with a semiclear periphery. (B, D) A clear visual axis appeared in the central pupil region.OM*-*type 3 (c): the cortex completely absorbed (missing). (A, C) The dilated pupil was covered by a white opacity before cataract surgery. A semiclear area in the periphery was seen. (B, D) A clear visual axis in the central pupil was achieved. OM-type 4 (d): a few blood vessels were observed. (A) The pupil was covered by a white opacity contained in a central Hemal net before cataract surgery. (B) A clear visual axis in the central pupil was achieved after cataract surgery. OM*-*type 5 (e): many blood vessels were observed. (A) The pupil was covered by a white opacity. A white opacity containing a central red mass was visible. There were many vessels in the lens. (B) A clear visual axis in the central pupil during cataract removal surgery was observed. (C) A posterior capsule opacification could be seen in the pupil, and a delayed hemorrhage after surgery was observed.

Congenital cataracts are relatively rare, and the systematic classification of congenital membranous cataract has not been established. Based on operating microscope observation, patients with membrane cataracts were classified as OM-type 1 through OM-type 5 as described below (Figure 2).

Congenital membrane cataract OM-type 1 is described as follows: the cortical and nuclear regions of the lens were partially absorbed as in cases 6, 7 (left eye), and 8 with type 1 cortical opacity. Anterior capsulorhexis was easily performed on these patients using a Klöti RF capsulotomy tip. A small amount of cortex could be found in the lens bag. The posterior capsule was always distended after the cortex was removed by I/A, so we had to very carefully avoid tearing the posterior capsule. The posterior capsule of 3 patients remained whole, and capsulectomy using a vitrectomy cutter was successful.

Congenital membrane cataract OM-type 2 is described as follows: the cortical and nuclear regions of the lens were mostly absorbed (missing) as in cases 2, 5, 7 (right eye), and 9. After anterior capsulorhexis was performed, the posterior capsule was also found to be cut in case 2, case 7 (right eye), and case 9. The posterior capsule was left whole after anterior capsulorhexis was performed in case 5, but the anterior and posterior capsules adhered closely in the center of the pupil, and we had to peel away the opaque membrane. We successfully performed a capsulectomy using a vitrectomy cutter.

Congenital membrane cataract OM-type 3 is described as follows: the cortical and nuclear regions of the lens were completely absorbed (missing) as in cases 3, 4, 10, and 11. The lenses were solid and compact. A curvilinear cut was made in the capsule with a Klöti RF capsulotomy tip. We kept a central flap that was linked at 6 o'clock with the rest of the capsule, and it was removed with a vitrectomy cutter.

Congenital membrane cataract OM-type 4 is described as follows: lenses were classified as OM-type 4 when they contained a few blood vessels, as in case 12. The lens in case 12 contained just a few vessels, and the periphery was transparent and the red reflex of the fundus could be seen. The classification was confirmed after the capsule was cut during the surgery. When capsulorhexis was performed, the blood vessels were cut. Bleeding was stopped by electrocoagulation using a Klöti RF capsulotomy tip.

Congenital membrane cataract OM-type 5 is described as follows: lenses were classified as OM-type 5 when many blood vessels were observed, as in case 1. The lens contained many blood vessels, and the red reflex of the fundus could not be observed by a surgical microscope. The classification was confirmed after the capsule was cut during the surgery. The vascular membrane was very thick and was cut with the Klöti RF capsulotomy tip layer by layer. Bleeding from the vessels occurred throughout the surgery. A Klöti RF capsulotomy tip was used to stanch the vessels. The lens material was successfully cut and aspirated using a vitrectomy cutter to achieve a clear visual axis, but the next day, we found a hemorrhage in the pupil. Two months after surgery, PCO and dislocated IOLs were found.

A comparison of the UBM and operating microscope examination classifications is shown in Table 3. In case 2 and cases 5–12, we could not confirm that the cataract was a congenital membranous cataract until the capsule was surgically removed. Therefore, the UBM examination prior surgery was useful to evaluate for congenital membranous cataract, to gain an optimal surgical outcome.

## 4. Discussion

Congenital membranous cataract is a rare type of cataract. Presently, there is no standardized surgery for it. Surgery is difficult because of the peculiar morphology of the cataract, which also makes it difficult to diagnose prior to the operation. Therefore, herein, we proposed a classification system for congenital membranous cataracts and recommend surgical methods based on our experience. We also explored the value of UBM examination for preoperative assessment the type of congenital membranous cataract. Some of our experiences treating congenital membranous cataracts are discussed below. In the present study, we reported surgical methods appropriate for the type of congenital membranous cataract, including anterior and posterior capsulorhexis using a Klöti RF capsulotomy tip. An RF capsulotomy tip was used to melt the capsular bag. There was no tearing when using a forceps or needle; simply gliding over the tissues with the capsulotomy tip was enough. This technique was suitable for patients with no fundus reflex. All the patients underwent successful operations and achieved a clear axis postoperatively.

The clinical features of congenital membranous cataracts are manifold. In this case series, we divided congenital membranous cataracts into two main types: vascular or avascular, with only the former containing vessels. The vascular type may be divided into those with few or many blood vessels. The patient of case 12 had a vascular membranous cataract with few vessels, which could be observed with a surgical microscope. The peripheral lens was transparent, and a red reflex of fundus could be seen. The lens opacity was confirmed as a thin membranous cataract, after the capsule was cut during the operation.

The patient of case 1 had a vascular membranous cataract with many vessels. In this case, the red reflex of fundus could not be observed by a surgical microscope. It was confirmed that the lens opacity was thick and rich in blood vessels in the absorbed cortex, after the capsule was cut during the operation.

A preoperative examination by UBM aids the diagnosis of congenital membranous cataract, as morphological characteristics can be observed directly. With UBM, 5 types of cataractous lens shapes were observed: thickened pizza-shaped, linear lens, flying saucer-shaped, dumbbell-like, and fusiform lens with a high-echo image. In every type, the UBM images allow assessment of the degree of cortex absorption, from partial to complete. In this study, a 5-type classification was obtained from 9 UBM images. In theory, with 9 more images, there would be more types. So the classification system for congenital membranous cataract was an open system. Once the new types of congenital membranous cataract were found with new UBM images, they could be added to the classification system.

At present, it is difficult to operate on congenital membranous cataract. Several papers of surgical procedures for congenital membranous cataract have been published [10, 11]. Our experience in this study indicates that different congenital membranous cataract types require different surgical procedures; we summarize 4 methods here. The first method is suitable for patients whose lens cortex is partially absorbed. In these patients, anterior capsulorhexis is performed easily with a Klöti RF capsulotomy tip; a little cortex remains in the lens bag. The posterior capsule is always hunched after the cortex is removed by I/A. Therefore, I/A must be done very carefully to avoid tearing of the posterior capsule. Finally, the posterior capsule is kept whole and a capsulectomy can be performed successfully using a vitrectomy cutter.

The second surgical method is suitable for patients whose lens cortex is mostly, but not completely, absorbed. The anterior and posterior capsules adhered closely in the center of the pupil, and the surgeon should peel away the opaque membrane and then remove it using a vitrectomy cutter. For these patients, a capsulectomy can be successfully performed using the vitrectomy cutter.

The third surgical method in congenital membranous cataract is appropriate for patients whose lens cortex is completely absorbed, with a membranous lens that is solid and compact. In these patients, the peripheral lens fibers are completely absorbed. When we perform a continuous curvilinear cut of the capsule with a Klöti RF capsulotomy tip, a central capsule flap is created. We keep the capsule adherent at 6 o'clock, with the rest of capsule removed by a vitrectomy cutter.

The fourth surgical method is suitable for patients with vascular membranous cataract. We found that capsulorhexis and electrocoagulation can be performed at the same time using the Klöti RF capsulotomy tip. The lens material could be cut with a vitreous cutter and aspirated.

For some patients with vascular membranous cataract, the vascular membrane is very thick and there are many blood vessels in the absorbed cortex. The membranous cataract can be cut, layer by layer, using a Klöti RF capsulotomy tip, and blood from the vessels stanched. Finally, the lens material is successfully cut and aspirated using the vitreous cutter. A clear pupil can also be achieved if delayed hemorrhages do not occur after the surgery. IOLs should be implanted cautiously to avoid hemorrhage.

UBM prior to congenital membranous cataract surgery is important for determining the optimal surgical procedure. In cases 2 and 5–12, we could not confirm that the congenital cataract was a congenital membranous cataract until the capsule was cut during the surgery. In some types of congenital membranous cataract, the cortex is partially absorbed. The anterior capsule and posterior capsule did not adhere and contained watery or emulsified-liked material in the central lens. The anterior and posterior capsules closed rapidly at the moment the anterior capsule was cut. During the capsulorhexis using a Klöti RF capsulotomy tip, the posterior capsule was cut at the same time. The diameter of the posterior capsule capsulorhexis is the same as that of the anterior capsule capsulorhexis, and we implanted the fixed loops of the IOL.

UBM images can be used to diagnose congenital membranous cataract before surgery. Owing to the preoperative UBM examination, the density of the congenital membranous opacity can be confirmed and a smooth surgical procedure can be achieved. In the meantime, the diameter of the capsulorhexis of the anterior capsule was designed to be the same as the posterior capsule, which is suitable for the IOL implantation. Therefore, the UBM examination prior to surgery is very useful to evaluate congenital membranous cataract to achieve an optimal surgical outcome.

## 5. Conclusion

Congenital membranous cataracts can be classified into several types based on the clinical characteristics of the lens. UBM played an important role in the preoperative assessment of the type of congenital membranous cataract. The surgical method chosen should depend on the type of congenital membranous cataract. Anterior and posterior capsulorhexis, capsulectomy with a Klöti RF capsulotomy tip and vitrectomy cutter, are effective surgical methods for treating congenital membranous cataract.

## Figures and Tables

**Table 1:** Different surgical methods for congenital membrane cataract.

Membrane features	Surgical procedures
(1) Solid and compact	Capsulotomy and excision of the membranous capsule and anterior vitrectomy were performed with vitreoretinal scissors.
(2) Thin, fibrous, and sticky	Both eyes were operated on using a routine pars plana lensectomy.
(3) Fibrotic and brittle	Using cystitome to capsulorhexis, the residual cortical matter was removed by I/A, and anterior vitrectomy was performed.
(4) Thin and compact	Scissors were used for capsulorhexis. The residual cortical matter was removed by I/A, and we performed an anterior vitrectomy.

**Table 2:** Characteristics of patients surgically treated for congenital membrane cataract.

	Case number											
	1	2	3	4	5	6	7	8	9	10	11	12
Gender (M/F)	F	F	F	M	M	M	M	M	F	F	M	F
Systemic disease	N	N	N	N	N	N	N	N	N	N	N	N
Time of diagnosis	Pre	Pre	Pre	Pre	Pre	Pre	Intra	Intra	Intra	Intra	Intra	Intra
Age at surgery (months)	5	6	8	6	32	17	71	25	16	71	7	4
Slit lamp	Y	Y	Y	Y	Y	Y	Y	Y	Y	Y	Y	Y
UBM	Y	Y	Y	Y	Y	Y	N	N	N	N	N	N
IOL implantation	+21 D	/	+25 D	/	OD: +16 D; OS: +16.5 D	/	/	/	/	+25 D	/	+26 D
Follow-up (months)	48	18	30	14	24	9	36	36	30	34	24	40
Complication OD	/	N	/	PCO	N	/	N	/	N	/	N	PCO
Complication OS	PCO	N	N	N	N	N	N	N	N	N	N	/

CMC: congenital membrane cataract; F: female; M: male; Y: yes; N: none; OD: right eye; OS: left eye; OU: both eyes; PCO: posterior capsule opacification; pre: preoperative; intra: intraoperative.

**Table 3:** Clinical characteristics of cataract morphology observed by UBM and operating microscopic examination by patient case.

UBM	UBM type	Operating microscopy	Congenital membranous cataract subtype
(1) OS: thickened pizza-shaped	1	Lens appeared white opaque with a red mass in the center. There were lots of vessels in the lens	Vascular membranous cataract (OM-type 4)
(2) OD: flying saucer-shaped	3	Pupil covered by a circular white opacity (right eye) and irregular white opacity (left eye) in the center before cataract surgery. A semiclear area in the periphery	Lens cortex was mostly absorbed (OM-type 2)
OS: pizza-shaped	1		
(3) OS: linear lens	2	Dilated pupil was covered by a white opacity before cataract surgery. A semiclear area in the periphery	Lens cortex was completely absorbed (OM-type 3)
(4) OU: linear lens	2	Dilated pupil was covered by a white opacity before cataract surgery. A semiclear area in the periphery	Lens cortex was completely absorbed (OM-type 3)
(5) OD: dumbbell-shaped	4	Dilated pupil was covered by a white opacity before cataract surgery. A semiclear area in the periphery	Lens cortex was mostly absorbed (OM-type 2)
OS: flying saucer-shaped	3		
(6) OS: fusiform	5	Pupil was covered by a daisy-like white opacity in the center before cataract surgery. A semiclear area in the periphery	Lens cortex was partially absorbed (OM-type 1)
